# Enterovirus D68 B3 clade strains are efficiently recovered from cDNA infectious clones in 293T cells and infect human spinal cord organoids

**DOI:** 10.1128/jvi.00450-26

**Published:** 2026-04-27

**Authors:** Jennifer E. Jones, Sarah Maya, Gal Yovel, Jessica Ciomperlik-Patton, Jennifer Anstadt, Megan Culler Freeman

**Affiliations:** 1 Department of Pediatrics, University of Pittsburgh School of Medicine 12317 , Pittsburgh, Pennsylvania, USA; 2 Department of Entomology, Texas A&M University 14736, College Station, Texas, USA; 3 Center for Vaccine Innovation and Access, PATH, Seattle, Washington, USA; University of Michigan Medical School, Ann Arbor, Michigan, USA

**Keywords:** tropism, infectious clone, organoid, enterovirus

## Abstract

Enterovirus D68 (EV-D68) is associated with respiratory disease in children. Between 2014 and 2018, EV-D68 infection was linked to peaks of a polio-like neurologic condition called acute flaccid myelitis (AFM), but currently circulating strains are not. We hypothesized that specific mutations within B3 clade strains impact neurovirulence. Few studies have investigated B3 clade strains, and none have recovered these viruses from cDNA infectious clones to understand how novel mutations in B3 clade strains impact neurovirulence. In this study, we examine the neurovirulence of recovered (r) B3 clade strains in relevant human cell culture and 3D organoid models. We demonstrate that rB3 clade strains replicate efficiently in the permissive RD cell line and in human respiratory epithelial cell lines, with BEAS-2B cells exhibiting greater permissivity than A549 cells. rB3 clade strains infect neural cells, including SH-SY5Y cells, a neuroblastoma cell line, and human spinal cord organoids, which model the cellular heterogeneity of the spinal cord. Immunofluorescence staining confirmed infection, with viral antigen colocalized with neurons. These findings are consistent with previous studies from our laboratory and others on the neurovirulence of clinical isolates of B3 clade strains. Overall, we provide a critical platform for rigorous interrogation of viral determinants of neurovirulence within the B3 clade.

**IMPORTANCE:**

Enterovirus D68 (EV-D68) can cause acute flaccid myelitis (AFM), a debilitating neurological condition of the spinal cord in children. Identifying viral determinants of EV-D68 neurovirulence is critical to understanding recent shifts in AFM prevalence; however, these investigations have been limited to a small subset of infectious clones distantly related to currently circulating B3 clade strains. In this study, we examine characteristics of recovered (r)EV-D68 strains from the dominant B3 clade. While all rEV-D68 replicate efficiently in a permissive cell line and in human respiratory epithelial cells, titers varied between strains in cultured neuroblastoma cells. Similarly, all B3 clade strains established infection in human spinal cord organoids, but replication varied between strains. Our study provides an essential platform for investigation into viral mutations within relevant B3 clade strains driving shifts in AFM prevalence.

## INTRODUCTION

Enterovirus D68 (EV-D68) is a non-enveloped member of the human enteroviruses in the *Picornaviridae* family (1). EV-D68 was first discovered in 1962 in oropharyngeal swabs from four children with pneumonia and bronchiolitis; one of these prototype strains is named “Fermon” (1). EV-D68 cases were rarely identified until 2005, when outbreaks of severe respiratory tract infections of EV-D68 were reported worldwide (2–4). This corresponded with viral diversification into three primary clades: A, B, and C (3–8).

EV-D68 is a reemerging pathogen associated with acute flaccid myelitis (AFM), a polio-like neurologic condition causing paralysis primarily in children (9, 10). Though the association between EV-D68 and AFM was first recognized in 2014, EV-D68 was later identified postmortem in a case from 2008 (11). In parallel with its novel association with AFM, EV-D68 continued to diversify into clades A1, A2/D, B1, B2, and B3 (10, 12–16). Biennial outbreaks of EV-D68-associated AFM recurred between 2014 and 2018 until viral transmission was disrupted in 2020 by nonpharmaceutical interventions implemented during the COVID-19 pandemic (17–23). EV-D68 circulation resumed in 2022, but was not associated with outbreaks of AFM for the first time since 2014 (23, 24). A comparison of clinical outcomes in 2018 and 2022 in Maryland indicated higher odds of hospital admissions and intensive care unit level of care in 2018 compared to 2022 (25). Whole-genome sequencing data further revealed a cluster of amino acid mutations associated with severe outcomes in 2018 (25). At the same time, the B3 clade grew increasingly dominant and continues to diversify into a new cluster (nextstrain.org) (25–27). Whether and how this new wave of diversification contributed to the stark decline in AFM prevalence is unclear.

cDNA infectious clones for EV-D68 were first engineered from the Fermon strain in 2018 by two research groups in China (28, 29). Sun et al. cloned the viral genome under a T7 promoter, whereas Pan et al. used a human RNA Pol I promoter. In both studies, Fermon virus was successfully recovered after transfection of 293T or RD cells directly with cDNA infectious clone (with co-transfection of T7 polymerase in the case of Sun et al.), but Pan et al. noted a PCR-induced mutation in the 5′ UTR that impaired replication (28, 29). Sun et al. later went on to apply their reverse genetics method to generate a cDNA infectious clone from a neurovirulent B2 clade strain (30). Two years later, cDNA infectious clones for Fermon as well as a limited number of B1 and B2 clade strains were generated in the US under a T7 promoter and deposited to the Biodefense and Emerging Infections Research Resources Repository (BEI Resources), making these the first widely available cDNA infectious clones for EV-D68 (31). Additional A1 and B3 clade cDNA infectious clones under a T7 promoter have since been deposited to BEI Resources by the Centers for Disease Control and Prevention, but to date, no published studies of neurovirulence determinants in EV-D68 leverage these relevant cDNA infectious clones. Therefore, despite the growing availability of EV-D68 cDNA infectious clones, the study of viral determinants of EV-D68 neuropathogenesis remains restricted to strains that are increasingly genetically distant from those currently circulating.

In this study, we examine the neurotropism of rEV-D68 from publicly available infectious clones for B3 clade strains. We systematically evaluated the recovery efficiency of rEV-D68 from infectious clones in RD and 293T cells. We demonstrate that while rEV-D68 recovery is achievable in all cell types, recovery in 293T cells is fastest and requires minimal reagents and source plasmid. This approach was well-suited to the recovery of diverse rEV-D68 strains, including the prototype Fermon and viruses representative of A1, B2, and B3 clades. We further report that while all rEV-D68 replicate efficiently in RD cells and in human respiratory epithelial cells, only viruses of the B2 and B3 clades can infect human neuroblastoma cells. Finally, we determined that human spinal cord organoids (hSCOs) are susceptible to infection with rB3 clade viruses. Altogether, we present a robust platform for dissecting neurovirulence determinants of EV-D68 from currently circulating neurotropic B3 clade viruses.

## RESULTS

### Recovery efficiency of EV-D68 from cDNA infectious clones is impacted by cell type, but not ratio of polymerase to infectious clone

**Fig 1:**
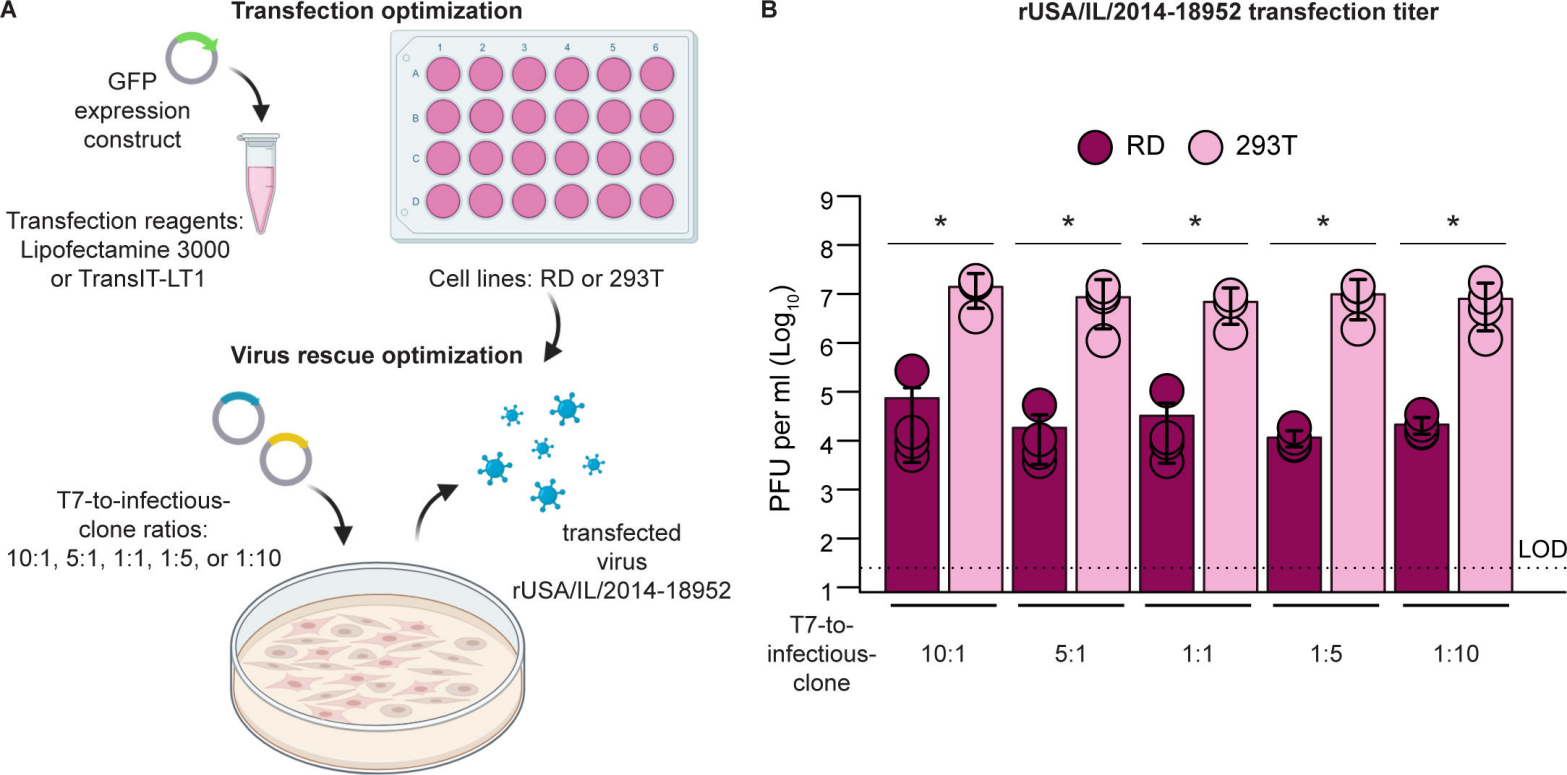
Recovery of rUSA/IL/2014-18952 from infectious clone is most efficient in 293T cells. (**A**) cDNA infectious clone rescue optimization schema. Transfection conditions were first optimized in each cell line with a GFP expression construct. Optimal transfection conditions were applied to virus rescue. RD or 293T cells were transfected with pUC19-EVD68_49131 and pCAGGS T7 at a ratio of 10:1, 5:1, 1:1, 1:5, or 1:10. Mock-transfected cells received transfection mixture without DNA. Cells were incubated at 33°C, 5% CO_2_. Cells were scraped from the dish and collected with supernatant. Created with Biorender.com. (**B**) Viral titers were quantified in recovered virus from each condition of (A) by plaque assay in RD cells. The means of two independently performed experiments are plotted as bars with standard deviation shown (*n* = 4 recovered viruses per condition). Statistical significance was determined by the Mann-Whitney *U*-test. Asterisks indicate *P* < 0.05.

Discrepancies in the literature in choice of cell line and conditions for delivery of viral genome have created ambiguity surrounding ideal recovery (r) conditions for rEV-D68 (28, 29, 31). Therefore, we began with a comprehensive investigation of EV-D68 recovery in two candidate cell lines: RD and 293T cells. To establish optimal recovery conditions, we first defined the transfection efficiency of each cell type. We optimized transfection efficiency in each cell line by titrating the concentration of a GFP expression plasmid, pcDNA 3.1 (+) CT-GFP, with either Lipofectamine 3000 or TransIT-LT1 (Fig. 1A). We scaled up transfection conditions for recovery of EV-D68 in each cell line with a well-characterized strain, rUSA/IL/2014-18952, since it is already readily recovered by published means (31, 32). We tested whether polymerase availability in RD and 293T cells impacts recovery efficiency using different ratios of T7 polymerase to cDNA infectious clone. The recovered virus readily induced observable CPE in 293T cells by 48 h and in RD cells by 72 h. Recovered virus was titered directly after transfection to assess viral yield immediately upon recovery. While a recent study reported that infectious virus is not readily detectable in 293T cells after transfection of the cDNA infectious clone (33), we found that both cell lines produced infectious virus in all conditions tested (Fig. 1B). Moreover, EV-D68 recovery was most efficient in 293T cells, with titers 1–3 logs higher than RD cells (*P* < 0.05 at all ratios of polymerase to cDNA infectious clone, Mann-Whitney *U*-test). Viral titers were highest in 293T cells, consistently exceeding 10^6^ PFU per mL. In contrast, the lowest viral titers (10^3^–10^5^ PFU per mL) were observed in RD cells. To assess the impact of the ratio of polymerase to cDNA infectious clone delivered, we performed linear regression on viral titers obtained from RD or 293T cells. The ratio of polymerase to cDNA infectious clone was not correlated with infectious virus titer in RD (*R*^2^ = 0.008, *F* (1, 18) = 0.14, *P* > 0.05) or 293T cells (*R*^2^ = 0.06, *F* (1, 18) = 1.12, *P* > 0.05). Thus, we concluded that recovery of EV-D68 is most efficient in 293T cells.

### B3 clade strains are efficiently recovered in 293T cells

**Fig 2:**
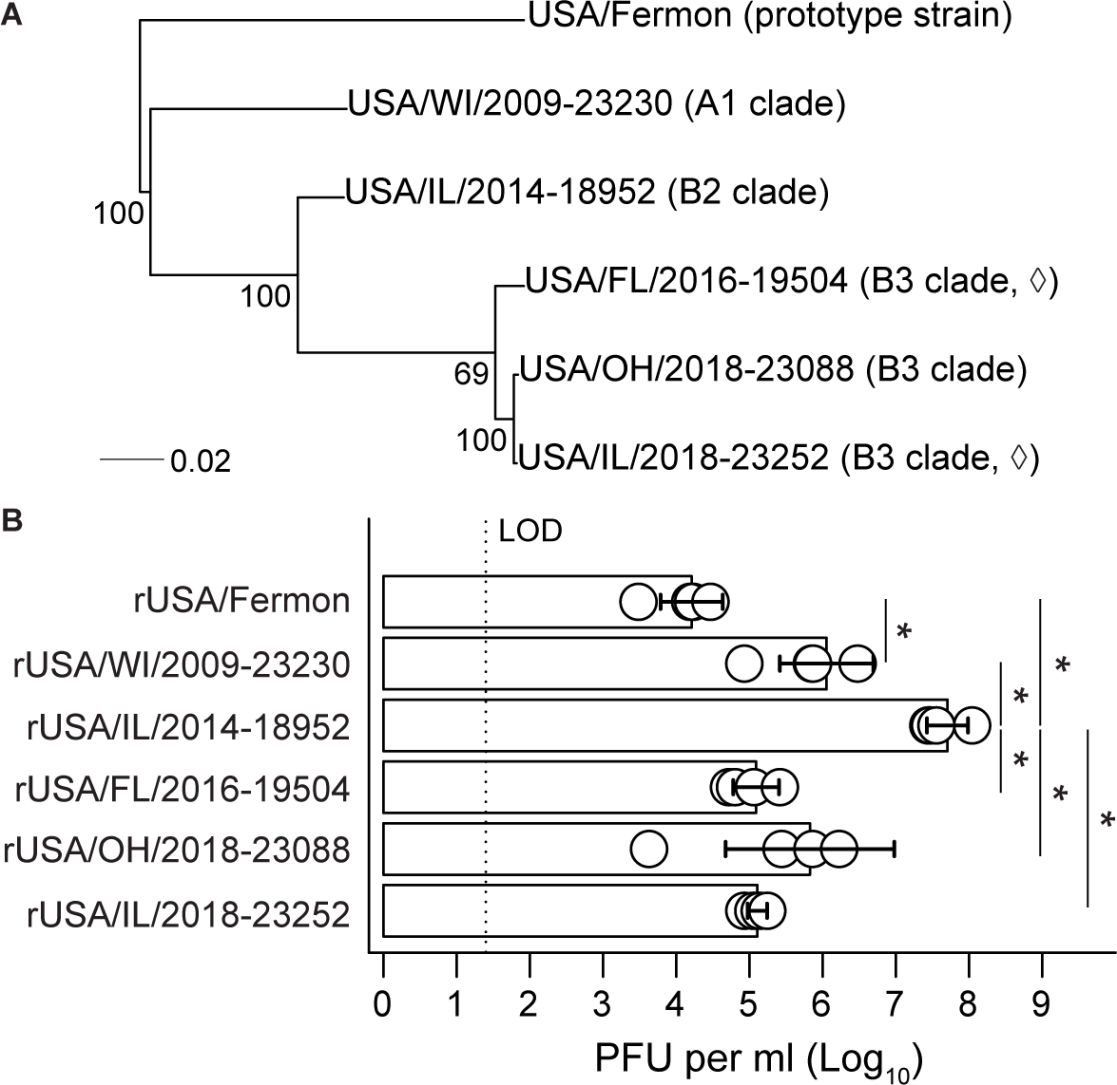
Contemporary EV-D68 infectious clones are efficiently recovered in 293T cells. (**A**) A maximum likelihood phylogenetic tree was reconstructed from VP1 sequences obtained from GenBank. Bootstrap values are for 1,000 trees. Scale bar indicates substitutions per site. (◊) denotes confirmed AFM. (**B**) The indicated rEV-D68 strains were rescued in 293T cells, and viral stocks were grown in RD cells. Viral titers were quantified by plaque assay in RD cells. The means of two independently performed experiments are plotted as bars with standard deviation shown (*n* = 4 recovered viruses per condition). Statistical significance was determined by one-way ANOVA with Tukey’s HSD post hoc test. Asterisks indicate adjusted *P* < 0.05.

Recovery of EV-D68 from some previously circulating clades is well-established (28–33), but to our knowledge, cDNA infectious clones for B3 clade strains remain unpublished despite their availability through BEI Resources. Therefore, we assessed whether the method we optimized for rUSA/IL/2014-18952, a B2 clade strain, facilitated recovery of virus from these cDNA infectious clones. cDNA infectious clones were available for USA/FL/2016-19504, USA/OH/2018-23088, and USA/IL/2018-23252. Two additional cDNA infectious clones were available through BEI Resources and were included in this study as controls as they were not expected to be neurotropic (34, 35): USA/Fermon (cDNA infectious clone deposited by reference 31) and USA/WI/2009-23230. To confirm clade assignments, we reconstructed a phylogenetic tree of VP1 sequences from all five of these strains as well as the neurovirulent B2 clade strain USA/IL/2014-18952. As expected, sequences from B3 clade strains USA/FL/2016-19504, USA/OH/2018-23088, and USA/IL/2018-23252 formed a cluster distinct from USA/IL/2014-18952 (Fig. 2A). Strains that preceded the 2014 association of EV-D68 with AFM, USA/Fermon and USA/WI/2009-23230, were distantly related to B2 and B3 clade strains.

We next tested our recovery strategy in all six cDNA infectious clones. Recently, Choi et al. determined that recovery of infectious enteroviruses from cDNA infectious clones in 293T cells is significantly improved with co-culture with a permissive cell line (33). In an analogous strategy, we consistently recovered infectious virus of 10^6^ PFU per mL or higher from all six cDNA infectious clones upon incubation of 293T cell transfection lysates with the permissive RD cell line (Fig. 2B). Titers for rUSA/IL/2014-18952 were significantly higher than all other recovered viruses, reaching 10^8^ PFU per mL (*P* < 0.05 in all pairwise comparisons, one-way ANOVA). B3 clade strain titers ranged from 10^4^ to 10^6^ PFU per ml and were not significantly different from the control strains rUSA/Fermon and rUSA/WI/2009-23230 (*P* > 0.05 in all pairwise comparisons). While not significantly different from B3 clade strains, titers for the recovered Fermon strain were lowest overall and significantly lower than rUSA/WI/2009-23230 (*P* < 0.05). Thus, our method permits efficient recovery of B3 clade EV-D68 from cDNA infectious clones.

### Recovered B3 clade strains replicate efficiently in the permissive RD cell line and human respiratory epithelial cell lines

**Fig 3:**
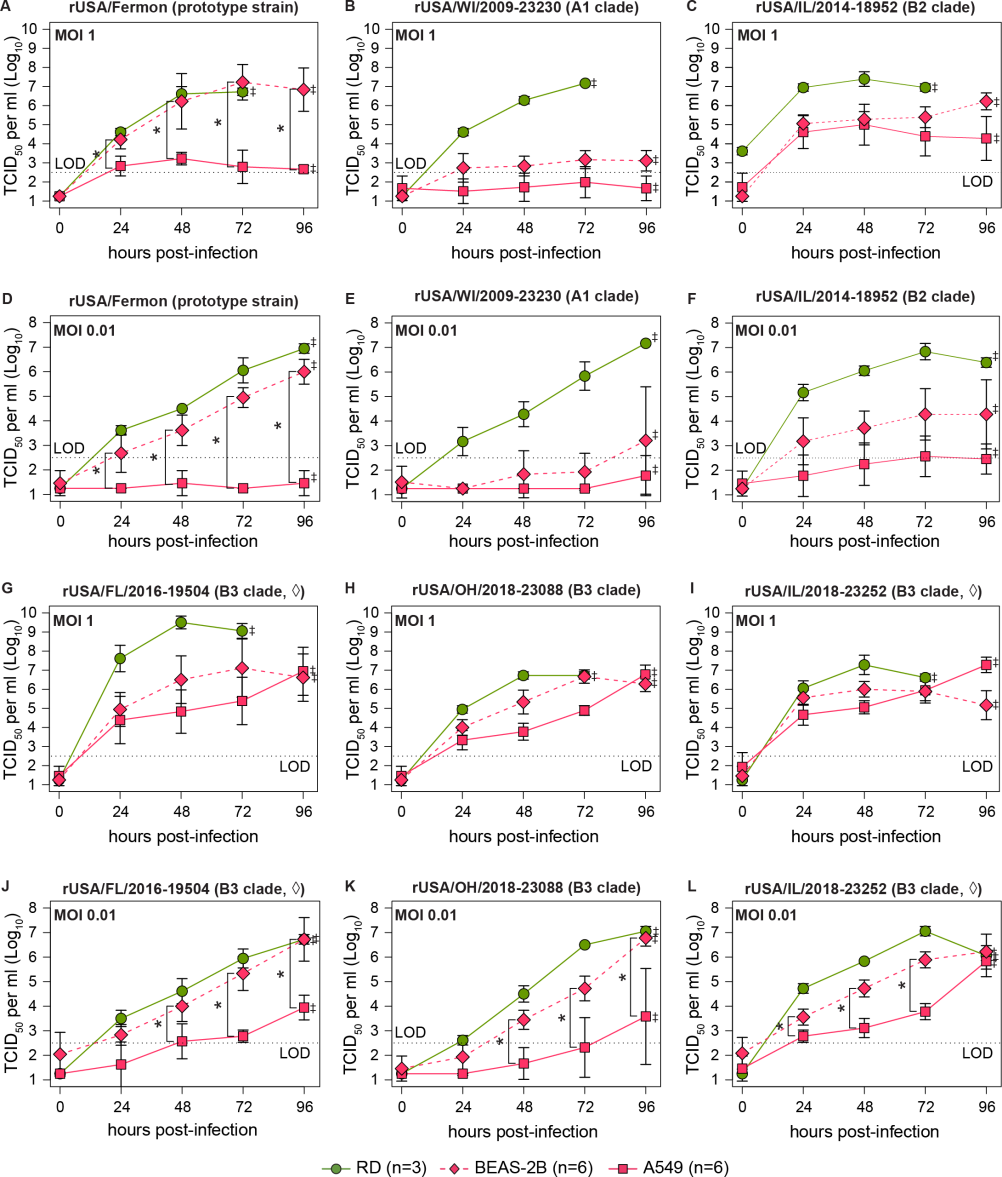
rEV-D68 strains replicate efficiently in parental RD and human respiratory epithelial cell lines. RD, BEAS-2B, and A549 cells were infected in triplicate with the indicated rEV-D68 strains at an MOI of 1 (**A–C and G–I**) or 0.01 (**D–F and J–L**). Supernatants were collected at the indicated time points. Endpoint titers represent combined cell lysate and supernatant, as denoted by (‡). (◊) denotes confirmed AFM. Viral titers were quantified by TCID_50_ assay on RD cells. The means of one (RD) or two (BEAS-2B, A549) independently performed experiments are plotted as a line graph with standard deviation indicated by error bars (*n* = 3 [RD] or 6 [BEAS-2B, A549] replicates per line). Statistical significance was determined by the Mann-Whitney *U*-test. Asterisks indicate *P* < 0.05.

At present, few studies have investigated the cellular tropism of B3 clade EV-D68 isolates (35, 36), and none have used viruses recovered from cDNA infectious clones. Therefore, we established the replication kinetics of this panel of rEV-D68 in susceptible cell types, beginning with the parental RD cell line on which our viral stocks were grown. As expected, all strains replicated efficiently in RD cells under single- and multi-cycle growth conditions (Fig. 3, green circles). Thus, our approach consistently produces replication-competent rEV-D68.

Since EV-D68 is most often detected in upper respiratory tract specimens (10, 37), we expected all recovered viruses to replicate efficiently in respiratory cells. Therefore, we examined replication kinetics in two human respiratory epithelial cell lines: BEAS-2B and A549. Each rB3 clade virus replicated efficiently in these cells at a multiplicity of infection (MOI) of 1 (Fig. 3G through I, diamonds or squares, respectively). Interestingly, all three rB3 clade strains replicated more efficiently in BEAS-2B cells over A549 cells at an MOI of 0.01 (Fig. 3J through L, diamonds vs squares; *P* < 0.05 at indicated time points, Mann-Whitney *U*-test). The B2 clade strain rUSA/IL/2014-18952 also replicated more efficiently in BEAS-2B cells than in A549 cells, although differences were not statistically significant (Fig. 3C and F, diamonds and squares). Differences between BEAS-2Bs and A549s were even more pronounced in the control strain, rUSA/Fermon, which replicated poorly in A549 cells even at high MOI (Fig. 3A, B and D, E, diamonds and squares; *P* < 0.05 at indicated time points). In fact, the only strain that exhibited robust replication in A549 cells at an MOI of 0.01 was the B3 clade strain rUSA/IL/2018-23252, although replication was significantly delayed between 24 and 72 hours post-infection (hpi) compared to BEAS-2B cells (Fig. 3L, squares; *P* < 0.05). The A1 clade strain rUSA/WI/2009-23230 was unable to replicate to high titer in either cell type at either MOI, though replication in RD cells was preserved (Fig. 3B and E). Overall, these data indicate that rB3 clade viruses replicate efficiently in respiratory epithelial cells in addition to the parental RD cell line, but replicative differences exist between human respiratory cell lines that are not clade-specific.

### Recovered B3 clade viruses efficiently infect neuroblastoma cells

**Fig 4:**
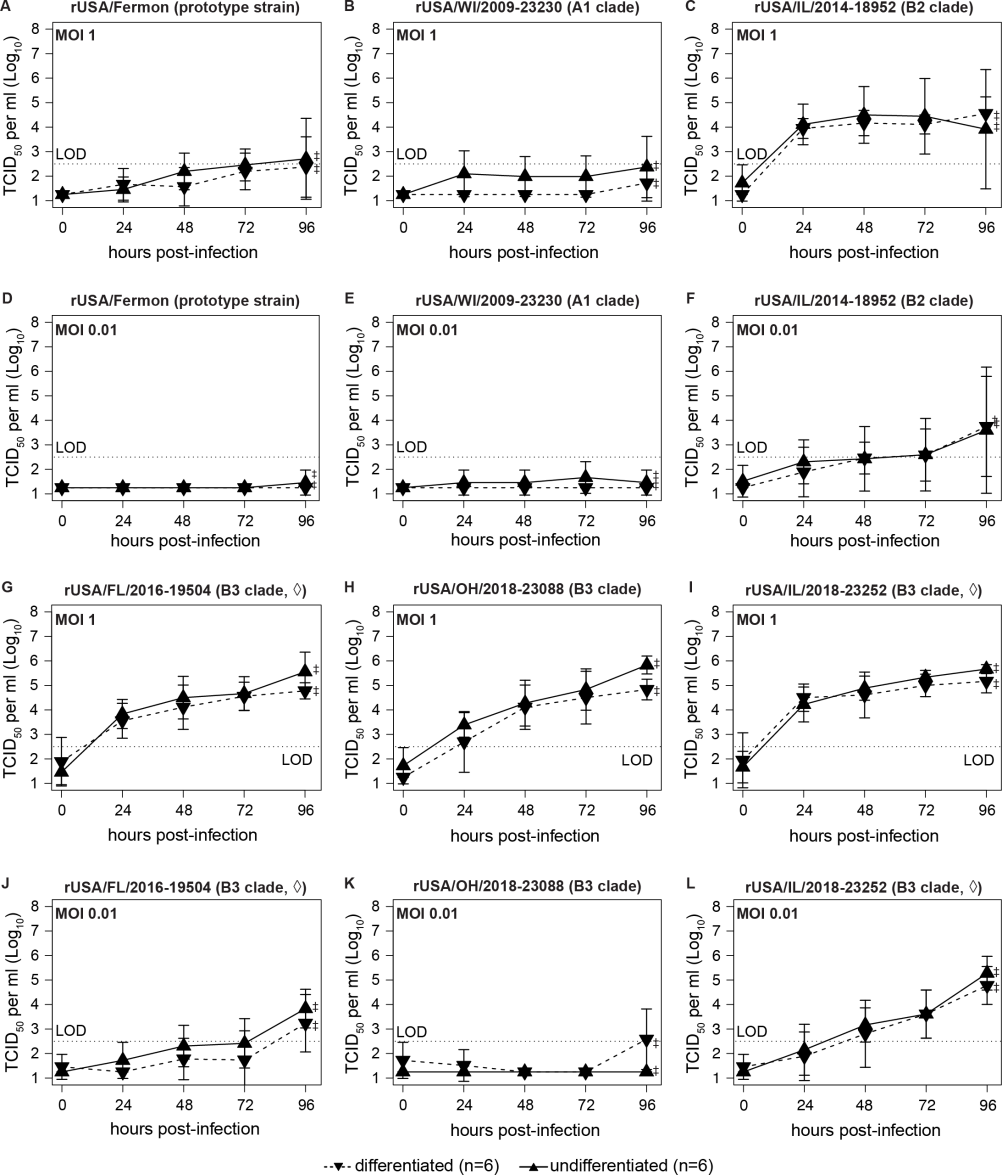
Recovered B3 clade strains replicate in neuroblastoma cells. SH-SY5Y cells were either differentiated into mature neuroblastoma cells (dashed lines) or maintained in growth medium (solid lines). After 72 h, cells were infected in triplicate with the indicated rEV-D68 strains at an MOI of 1 (**A–C and G–I**) or 0.01 (**D–F and J–L**). Supernatants were collected at the indicated time points. Endpoint titers represent combined cell lysate and supernatant, as denoted by (‡). (◊) denotes confirmed AFM. Viral titers were quantified by TCID_50_ assay on RD cells. The means of two independently performed experiments are plotted as a line graph with standard deviation indicated by error bars (*n* = 6 replicates per line).

EV-D68 has been associated with large outbreaks of AFM since 2014 (9, 10). Some, but not all, rEV-D68 from previously circulating B1 and B2 clades can invade the CNS in mouse models (30–32, 38), but rEV-D68 from the currently circulating B3 clade has not been evaluated for neurotropism. Therefore, we examined whether our panel of rB3 clade strains is neurotropic in cell culture models. We first explored replication kinetics of our B3 clade viruses in the human neuroblastoma SH-SY5Y cell line, an established model for EV-D68 neurotropism (34, 36). Infectious virus was not consistently detected in SH-SY5Y cells infected with control rEV-D68 strains at the MOIs tested (Fig. 4A, B and D, E), in agreement with previous literature suggesting that neurotropism in EV-D68 is rare in strains isolated prior to 2014 (9, 10, 34–37). Infectious virus was detectable beginning at 24 hpi at an MOI of 1 for the B2 clade rUSA/IL/2014-18952 strain (Fig. 4C, solid line). Similarly, all B3 clade viruses replicated efficiently in SH-SY5Y cells at an MOI of 1 (Fig. 4G through I, solid lines). Surprisingly, each B3 clade strain achieved a peak titer of 10^5^–10^6^ TCID_50_ per mL. At an MOI of 0.01, differences between neurotropic strains emerged. Compared to replication at an MOI of 1, replication was delayed in all four strains, with the B3 clade rUSA/OH/2018-23088 strain only producing detectable virus at the experimental endpoint of 96 hpi and only when cells were differentiated (Fig. 4F and J through L). Only rUSA/IL/2018-23252 exhibited sustained logarithmic growth in these cells at this MOI (Fig. 4L). We quantified replication differences between neurotropic strains by performing linear regression on viral titers in SH-SY5Y cells at an MOI of 0.01. The regression model explained a statistically significant proportion of overall replication differences in SH-SY5Y cells (*R*^2^ = 0.56, *F* (9, 770) = 108.44, *P* < 2.2 × 10^−16^). Consistent with our observations, MOI was statistically significant (*P* < 2.2 × 10^−16^). Neither of the control rEV-D68 was statistically significant, but differences between neurotropic strains were significant (*P* < 2.2 × 10^−16^ for rUSA/IL/2014-18952, rUSA/FL/2016-19504, and rUSA/IL/2018-23252; *P* = 5.7 × 10^−14^ for rUSA/OH/2018-23088). Overall, these data demonstrate that rB3 clade strains efficiently infect cultured neuroblastoma cells but differ in their capacity for continuous replication under low MOI conditions.

### Human spinal cord organoids are susceptible to rB3 clade virus infection

Our studies thus far suggest that rB3 clade strains can infect and replicate in cells of the neuroblastoma SH-SY5Y cell line. However, the spinal cord is comprised of intricate neural circuits with over twenty neuron subclasses and glial cells (39). We sought to determine whether rB3 clade viruses could establish infection in a more complex model of the spinal cord. Therefore, we further investigated neurotropism of this panel of rEV-D68 strains in three-dimensional spinal cord (3DiSC) organoids. Under 3DiSC differentiation conditions, human spinal cord organoids (hSCOs) are differentiated from human inducible pluripotent stem cells for 14 days and develop a continuous dorsal neuroepithelial tissue architecture (39). These hSCOs recapitulate more of the cellular heterogeneity found in the spinal cord, and we have previously shown that they are susceptible to clinical EV-D68 isolates from contemporary clades, including the B3 clade (35).

**Fig 5:**
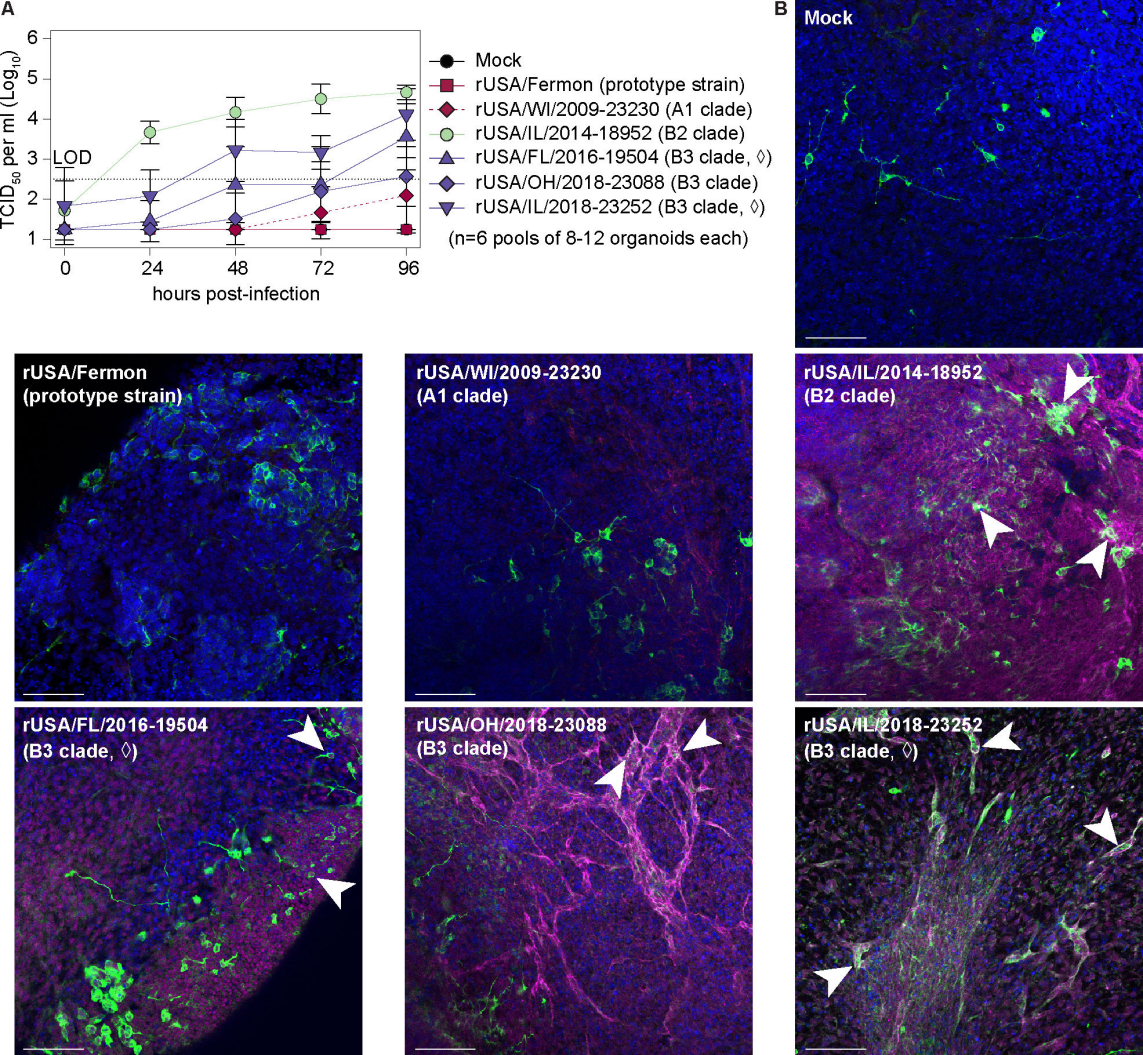
Human spinal cord organoids are susceptible to rB3 clade strain infection. Human inducible pluripotent stem cells were differentiated into three-dimensional spinal cord (3-DiSC) organoids for 14 days. Pools of 8–12 organoids were either mock-infected or infected with rEV-D68 at 10^6^ PFU per pool in triplicate. (◊) denotes confirmed AFM. (**A**) Viral titer in supernatant was quantified by TCID_50_ assay on RD cells. The means of two independently performed experiments are plotted as line graphs with standard deviation indicated by error bars (*n* = 6 organoid pools with 48–72 total organoids per line). (**B**) Organoids were fixed at 96 hpi and stained by immunofluorescence for 4′,6-diamidino-2-phenylindole (DAPI) in blue, a neuronal marker class III β-tubulin (Tuj1) in green, or VP1 in magenta. White arrowheads denote colocalization between VP1 and Tuj1. Scale bars, 100 μm.

We first examined growth kinetics of each rEV-D68 strain in hSCOs at day 14 post-differentiation, when neurons are present but still relatively immature. Consistent with our results in SH-SY5Y cells and with clinical isolates (35), the control strains rUSA/Fermon and rUSA/WI/2009-23230 did not consistently produce infectious virus throughout the duration of this experiment (Fig. 5A, red diamonds and squares). Similarly to what we observed in SH-SY5Y cells at high MOI, the B2 clade rUSA/IL/2014-18952 strain exhibited sustained replication in hSCOs, with a peak infectious virus titer of 10^5^ TCID_50_ per mL (Fig. 5A, green circles). In a pattern analogous to replication in SH-SY5Y cells under low MOI conditions, both rUSA/FL/2016-19504 and rUSA/IL/2018-23252 replicated efficiently in hSCOs, with infectious virus first detected by 48 hpi (Fig. 5A, standard or inverse purple triangles). Replication of rUSA/OH/2018-23088 was markedly delayed in hSCOs, in a pattern consistent with that observed in differentiated SH-SY5Y cells at low MOI (Fig. 5A, purple diamonds). To examine replication differences between strains in hSCOs, we fitted replication data to a regression model. A linear model captured a statistically significant proportion of replication differences in hSCOs (*R*^2^ = 0.68, *F* (7, 202) = 61.02, and *P* < 2.2 × 10^−16^). Within this model, the B2 clade rUSA/IL/2014-18952 strain (*P* < 2 × 10^−16^), and two B3 clade strains, rUSA/FL/2016-19504 (*P* = 7.13 × 10^−5^), and rUSA/IL/2018-23252 (*P* = 1.99 × 10^−12^), were statistically significant. Neither the control rEV-D68 strains nor the B3 clade rUSA/OH/2018-23088 strain was significant. Based on these findings, we conclude hSCOs are permissive to rB3 clade strains.

To confirm that all rB3 clade strains established infection in hSCOs, we additionally performed immunofluorescence staining of hSCOs for VP1, the viral capsid protein. Growth kinetics of rEV-D68 strains in hSCOs mirrored replication kinetics in differentiated SH-SY5Y neuroblastoma cells. Therefore, we examined whether VP1 in infected hSCOs colocalized with class III β-tubulin (Tuj1), an early neuronal differentiation marker (40). We collected hSCOs at 96 hpi, when infectious virus was detected for all rB2 and rB3 clade strains and processed them for immunofluorescence. Tuj1 staining was readily detected in hSCOs at this time point (Fig. 5B, green). Minimal to no VP1 staining was observed in hSCOs that were mock-infected or infected with control strains; in contrast, robust VP1 staining was detected in all hSCOs infected with rB2 and rB3 clade strains (Fig. 5B, magenta). Modest VP1 staining was observed for rWI/2009-23230, but no infectious virus was detected for this strain throughout the time course (Fig. 5A, red diamonds). Conversely, VP1 staining was prominent in hSCOs infected with rUSA/FL/2016-19504 and rUSA/OH/2018-23088, despite the limited replication seen for these strains in hSCOs overall (Fig. 5A, purple triangles and diamonds). Colocalization of VP1 with Tuj1 was seen in all B2 and B3 clade rEV-D68 strains (Fig. 5B, arrowheads). Altogether, these data demonstrate that hSCOs are susceptible to rB3 clade virus infection.

## DISCUSSION

Polymerases encoded by RNA viruses lack proofreading enzymes, leading to the replication infidelity and high mutation frequencies characteristic of viruses like EV-D68 (reviewed in references 41, 42). For such viruses, a robust reverse genetics system is not only integral to studies of viral pathogenesis but can greatly improve reproducibility of experimental findings between independent research groups whose lab stocks of clinical isolates may differ. While cDNA infectious clone technology for RNA viruses has existed for decades, a comprehensive reverse genetics system for multiple clades of EV-D68 is lacking. This has contributed to controversial findings for EV-D68, such as conflicting reports of neurotropism in clinical isolates for the prototype strain Fermon when propagated in different laboratories (9, 10, 34–37, 43). Here, we demonstrate for the first time, reliable recovery of infectious virus from EV-D68 cDNA infectious clones of the prototype strain Fermon and representative A1, B2, and B3 clade viruses (Fig. 2) by direct transfection of cDNA infectious clone and T7 polymerase plasmids into 293T cells, alleviating this limitation on further studies of EV-D68 pathogenesis and enhancing reproducibility of findings between research groups. Transfection efficiency played a key role in the choice of cell line and likely contributes to this dearth of research utilizing cDNA infectious clones for EV-D68. Even after extensive optimization of different commercial transfection reagents and plasmid DNA at a range of dosages, fewer than half of RD cells took up DNA, which undoubtedly impacts DNA delivery and the number of rounds of viral replication required to produce infectious virus. In contrast, maximal transfection efficiency of 293T cells was achieved with a fraction of the amount of plasmid DNA and transfection reagent and produced visible CPE 24 h sooner than in RD cells. The characteristics of each rEV-D68 strain analyzed in this study are summarized in Table 1.

**TABLE 1:** Summary of growth characteristics of rEV-D68 strains^*a*^^,^^*b*^^,^^*c*^^,^^*d*^^,^^*e*^

Clade	Strain	Parental cell line	Respiratory epithelial cell lines		Neuroblastoma cell line		Spinal cord organoid	
					UN	DIFF		
		RD	BEAS-2B	A549	SH-SY5Y		hSCO	
		Replication						VP1
Prototype	rUSA/Fermon	+++	+++	+	–	–	–	–
A1	rUSA/WI/2009-23230	+++	+	–	–	–	–	+
B2	rUSA/IL/2014-18952	+++	+++	++	+	++	+++	+++
B3	rUSA/FL/2016-19504	+++	+++	++	++	++	+	++
B3	rUSA/OH/2018-23088	+++	+++	++	++	++	–	+++
B3	rUSA/IL/2018-23252	+++	+++	+++	+++	+++	+	+++

^*a*^ –, no replication/VP1 observed.

^*b*^ +, modest replication at an MOI 1 and poor replication at an MOI of 0.01 (cell lines); any replication/VP1 observed (hSCO).

^*c*^ ++, robust replication observed at an MOI of 1 only (cell lines); sustained replication or VP1 (hSCO).

^*d*^ +++, robust replication observed at an MOI of 1 and 0.01 (cell lines); robust replication/VP1 (hSCO).

^*e*^ Growth kinetics in human cell lines (RD, BEAS-2B, A549, or SH-SY5Y) or human iPSC-derived spinal cord organoids (hSCO) are summarized under the Replication heading. VP1 expression in hSCOs stained by immunofluorescence are summarized under the VP1 heading. UN, undifferentiated; DIFF, differentiated.

While nearly all rEV-D68 strains replicated efficiently in respiratory epithelial cells, replication was highly constrained in A549 cells compared to BEAS-2B cells (Fig. 3). The inverse relationship was found for respiratory syncytial virus (RSV), which more effectively replicates in A549 cells than BEAS-2B cells (44). While both cell lines were initially isolated from human lungs, A549 cells are classified as alveolar type II basal epithelial cells, originally cultured from tissue of an explanted lung adenocarcinoma tumor (45). BEAS-2B cells were instead cultured from autopsy tissue in a non-cancerous normal bronchial epithelium and later immortalized with SV40 T antigen (46). During infection with RSV, A549 cells express high levels of IFN-β-, IFN-λ-, and NF-κB-inducible proinflammatory cytokines, whereas interferon-stimulated genes, pattern recognition receptors, and other antiviral genes are upregulated in BEAS-2B cells (44). Given that replication differences between these two cell lines were frequently exacerbated at lower MOI in our study, infection with rEV-D68 likely induces greater antiviral signaling in A549 cells than in BEAS-2B cells. Thus, discrepancies in biological origin, subsequent propagation, and antiviral responses of these cell lines could contribute to the differences observed in viral tropism of rEV-D68.

This study leveraged two human models of the CNS to investigate neurotropism of rB3 clade strains. rB3 clade viruses were neurotropic in both models, but differed from the paralytogenic B2 clade strain rUSA/IL/2014-18952 (Fig. 3 and 4). Replication of rUSA/IL/2014-18952 was sensitive to MOI in SH-SY5Y cells but was highly efficient in hSCOs, which might suggest a greater availability of susceptible cell types in this multicellular model of the human spinal cord compared to SH-SY5Y cells. Given that hSCOs are both differentiated and highly heterogeneous (39), replication of this strain may be mediated by differentiated neurons or other non-neuronal cell lineages. In contrast, replication of all B3 clade strains in hSCOs mirrored replication in SH-SY5Y cells under low MOI conditions, suggesting fewer cell types susceptible to B3 clade viruses relative to the B2 clade rUSA/IL/2014-18952 in hSCOs after 14 days of differentiation. It is possible that more mature hSCOs may be more susceptible to rB3 clade viruses. However, it is critical to note that neurotropism is defined here as the capacity for infection (assessed by VP1 staining) and sustained replication (assessed by production of infectious virus in culture supernatant) in human models of the CNS. Moreover, MOI cannot be reliably calculated in an organoid comprised of many different cell types, as the number of susceptible cells is unknown. In a recent study from our group, we leveraged single-cell RNA sequencing to define the transcriptional profile of two clinical isolates of EV-D68, the B2 clade USA/IL/2014-18952 strain examined in this study and a B3 clade strain USA/MA/2018-23089, in hSCOs (47). These clinical isolates differed in both cellular tropism within hSCOs and host transcriptional responses to infection, with the B3 clade strain exhibiting greater tropism for proliferating astrocytes and oligodendrocyte progenitor cells. Infection with the B3 clade strain enriched proliferative and biosynthetic transcription programs in a strain-specific manner, likely driven by direct infection of proliferating astrocytes. These studies were performed in hSCOs at 24 days post-differentiation, consistent with our hypothesis that B3 clade strains may be more sensitive to maturation. Functional outcomes such as transcriptional responses or neuronal viability were not assessed in the current study, but our previous studies suggest that these may differ between strains.

Ongoing viral evolution likely contributes to the differential susceptibility we observed between strains. Unfortunately, a systematic investigation of neurovirulence determinants between the prototype strain USA/Fermon and a neurovirulent strain has, to our knowledge, never been performed. However, two previous studies using murine models mapped viral determinants of CNS infection and paralysis to the capsid proteins VP1 and VP3 (31, 32). Recent work from our group leveraged our system for recovery of infectious virus from cDNA infectious clones to reconstruct these published neurovirulence determinants, confirming that VP3 I88V and VP1 L1I/N2D/T98A/E283K attenuate neurotropism of rUSA/IL/2014-18952 in hSCOs (48). These previously published determinants, however, do not explain the altered neurotropism phenotype for the strains in our study without replication in our neural models. The VP3 V88 neurovirulence determinant is rare (48) and does not occur in either of the control strains in our study. In USA/Fermon, the prototype strain, VP1 has a serine encoded at position 1, an asparagine encoded at position 2, and an aspartic acid encoded at position 283. USA/WI/2009-23230 encodes the neurovirulent VP1 L1 and E283, and both control strains encode the neurovirulent VP1 T98, suggesting that additional neurotropism determinants exist elsewhere to account for the impaired CNS tropism we observed.

Given that a majority of currently circulating EV-D68 strains are B3 clade, a deeper understanding of B3 clade genetic determinants is necessary. While all rB3 clade strains in the current study could infect human models of the CNS, replication kinetics differed, particularly when compared to the B2 clade rUSA/IL/2014-18952. Consistent with their pattern of CNS tropism in the present study, none but rUSA/FL/2016-19504 encodes previously identified neurovirulence determinants. rUSA/FL/2016-19504 encodes only VP1 A98, which may account for some of the modest neurotropism observed in this virus. The B3 clade strains examined in the current study exhibit 87% pairwise identity to USA/IL/2014-18952, with amino acid substitutions found across the viral genome in VP4 (V65A), VP2 (G74T, N138T, E151A), VP1 (A6G), 2A (K25R, S84N, V144A), 2C (N34T, A102M, D273G, T277V or T277A), 3C (N50D, K55R), and 3D (T105S, S166N). While these mutations may not impact the capacity for infection of the CNS, they may contribute to functional outcomes such as the proliferative and biosynthetic transcriptional profile we observed in a clinical B3 isolate from 2018 (47). Moreover, B3 clade strains continue to evolve, and substitutions have been described in currently circulating B3 clade strains (VP1 D2E and T98A, 2A T57A and N84S, 3A I8V, and 3D I212M) that were associated with a shift towards less severe respiratory disease in 2022 (25). Ultimately, the ability to recover infectious virus from circulating B3 clade rEV-D68 strains has provided exciting new avenues for investigation into novel viral determinants of AFM.

## MATERIALS AND METHODS

### Cells

Human embryonic kidney (HEK) 293T cells (ATCC CRL-3216), human adenocarcinoma lung epithelial A549 cells (ATCC CCL-185), human rhabdomyosarcoma (RD) cells (ATCC CCL-136), human lung bronchial epithelial BEAS-2B cells (ATCC CRL-3588), and human neuroblastoma SH-SY5Y cells (ATCC CRL-2266) were obtained from ATCC. Human iPSCs were obtained from STEMCELL Technologies (SCTi003-A). A549, 293T, RD, and BEAS-2B cells were maintained in Dulbecco’s modified Eagle’s medium (DMEM, Corning) supplemented with 10% fetal bovine serum (FBS, Biowest) and 1% penicillin/streptomycin (Gibco). SH-SY5Y cells were maintained in EMEM supplemented 1:1 with Ham’s F12 nutrient mix (Gibco), 10% FBS, 1% sodium pyruvate, 1% nonessential amino acids, and 1% penicillin/streptomycin. Where specified, SH-SY5Y cells were differentiated into mature neurons by the reduction of the FBS concentration in the culture medium to 3% and the addition of 100 nM retinoic acid (Tocris) for 72 h, as previously described (34). Human iPSCs were maintained in mTeSR Plus medium (STEMCELL Technologies) in flasks coated with 150 µg/mL Cultrex (R&D Systems). All cells were routinely tested for mycoplasma contamination with a PCR-based detection kit either in-house (Sigma) or at the University of Arizona Genetics Core (Applied Biological Materials Inc.). Cells were maintained in sterile cell culture incubators at 37°C, 5% CO_2_ unless otherwise stated. Low-passage cultures were used to ensure the purity of the culture.

### Cell line authentication

All cell lines used in this study were authenticated by short tandem repeat (STR) profiling. Genomic DNA was extracted from cells using the DNeasy Blood and Tissue Kit (Qiagen) following the manufacturer’s guidelines and shipped to the University of Arizona Genetics Core for STR profiling. Briefly, genomic DNA was genotyped for 15 autosomal STR loci and amelogenin (X/Y) with the Powerplex 16HS PCR kit (Promega). Positive control (gDNA 2800M, Promega) and negative control (water) were also amplified to confirm the accuracy of allelic calls and that reactions were free of contaminating genetic material. PCR products were separated by capillary electrophoresis using an AB 3730 DNA Analyzer. Applied Biosystems Internal Size Standard GeneScan500-LIZ was used to standardize allele size calls for each sample. Samples were run on a 36 cm capillary array (Applied Biosystems). Electropherograms were analyzed and allelic values assigned using Soft Genetics, Gene Marker Software Version 3.0.1. Alleles were matched to the STR profile recorded in the Deutsche Sammlung von Mikroorganismen und Zellkulturen (DSMZ) Leibniz Institute database (when reference profile is available). An American National Standards Institute (ANSI) standard of a minimum 80% match threshold indicates a shared genetic history.

### Plasmids and transfection optimization

The following EV-D68 cDNA infectious clones were obtained through BEI Resources, National Institute of Allergy and Infectious Diseases (NIAID), National Institutes of Health (NIH): rUSA/Fermon was recovered from pUC19-R-Fermon (NR-52375), rUSA/WI/2009-23230 from pUC-R23230 (NR-52377), rUSA/IL/2014-18952 from pUC19-EVD68_49131 (NR-52011), rUSA/OH/2018-23088 from pUC-R23088 (NR-52379), rUSA/IL/2018-23252 from pUC-R23252 (NR-52380), and rUSA/FL/2016-19504 from pUC-R19504 (NR-52378).

Sequences of plasmid stocks of infectious clones were verified by whole-plasmid, long-read sequencing (Oxford Nanopore, Plasmidsaurus). The following discrepancies were found between cDNA infectious clone sequences and their GenBank counterparts: pUC-R19504 and pUC-R23088, 2A S11F; pUC-R23088 and pUC-R23252, 3D D43N. The pCAGGS T7 opt plasmid was obtained through Addgene (#65974).

Transfection conditions were empirically determined for RD and 293T cells. Briefly, each cell type was grown in tissue culture plates. Cells were transfected with a pcDNA 3.1 (+) CT-GFP reporter construct (Invitrogen) and either TransIT-LT1 transfection reagent (Mirus Bio) or Lipofectamine 3000 (Invitrogen) at a range of concentrations according to the manufacturer’s specifications. GFP expression was monitored at 24–72 h post-transfection using an EVOS FL Auto 2 cell imaging system (Invitrogen). Transfection conditions yielding maximal GFP expression and minimal toxicity were selected and applied to virus recovery. Lipofectamine 3000 was selected for RD cells, and TransIT-LT1 transfection reagent was chosen for 293T cells.

### Virus recovery optimization

Virus recovery conditions were empirically determined for RD and 293T cells. Briefly, each cell type was grown in tissue culture dishes. Transfection conditions for each cell type were scaled up from the GFP transfection experiment and applied to pUC19-EVD68_49131 to recover rUSA/IL/2014-18952. Cells received pCAGGS T7 expression construct at the following ratios to cDNA infectious clone: 10:1, 5:1, 1:1, 1:5, or 1:10. Mock-transfected cells received transfection mixture without DNA. Transfections were performed according to the manufacturer’s specifications, and cells were incubated at 33°C, 5% CO_2_ until collection. Plaque assays were performed on recovered virus, and recovery conditions were selected based on these titers.

The full panel of recovered viruses, including a fresh stock of rUSA/IL/2014-18952, was recovered from infectious clones in 293T cells. Cells were grown on tissue culture dishes. Transfection mixtures were supplied with pCAGGS T7 expression construct and the indicated cDNA infectious clone at a ratio of 10:1. Transfections were performed with TransIT-LT1 transfection reagent (Mirus Bio) according to the manufacturer’s specifications. Mock-transfected cells received transfection mixture without DNA. Cells were incubated at 33°C, 5% CO_2_ until collection.

Viral stocks were obtained by incubation of transfected lysates on RD cells. Briefly, RD cells were grown on tissue culture dishes. Half of the transfection lysate was added to cell monolayers, and cells were rocked at room temperature for 1 h to adsorb virus. Mock-infected cells received cell culture media. Inocula were aspirated from dishes, and cell growth medium was replenished. Cells were incubated at 33°C, 5% CO_2_ until collection. Viral stocks were harvested and clarified by ultracentrifugation at 12,000 rpm for 10 min at 16°C and stored at −80°C.

### Phylogenetics

Sequences were read into R (version 4.3.2) and analyzed with the DECIPHER (version 2.30.0), ape (version 5.7-1), and phangorn (version 2.11.1) packages. A maximum-likelihood tree was reconstructed from a multiple sequence alignment and rooted to the prototype strain (USA/Fermon). A general time-reversible model was selected based on log likelihood and Akaike information criterion (AIC). Bootstrapping was performed with 1,000 trees.

### Growth kinetics in human cell lines

RD, A549, BEAS-2B, and SH-SY5Y cells were grown in tissue culture plates. Half of the SH-SY5Y cells were differentiated into mature neurons. rEV-D68 was diluted to an MOI of 1 or 0.01 in PBS containing calcium and magnesium (Corning). Cells were inoculated in triplicate with the indicated strain or mock-infected with diluent. Inocula were adsorbed onto cells at room temperature for 1 h with rocking. After 1 h, the inoculum was aspirated, cells were washed three times with PBS without calcium/magnesium (HyClone), and growth medium was added to cells. An aliquot was taken from each well and designated 0 hpi. Cells were incubated at 33°C, 5% CO_2_ for the course of infection, with supernatants collected at 24, 48, 72, and 96 hpi. At the endpoint of the assay, cells were scraped and harvested with their respective supernatants. Samples were stored at −80°C.

### Differentiation and propagation of human spinal cord organoids

3-DiSC hSCOs were differentiated from iPSC cells as previously described (35). Briefly, iPSC cells were dissociated into 96-well round-bottom low-adhesion plates using Accumax (Sigma). Cells were seeded at a density of 9,000 cells per well. Cells were plated in differentiation medium for 14 days, with media replenished every 3 days. After 14 days, organoids were transferred to a tissue culture plate and cultured in suspension in N2B27 medium (DMEM/F-12 [Gibco], neurobasal medium [Gibco] [1:1], 0.5% [vol/vol] N2 supplement [ThermoFisher] and 1% [vol/vol] B27 supplement without vitamin A [Gibco]) supplemented with 1 mM l-glutamine (ThermoFisher), 0.1 mM β-mercaptoethanol, 0.5 µM ascorbic acid, 10 ng/mL brain-derived neurotrophic factor (BDNF, STEMCELL Technologies), 10 ng/mL glial cell line-derived neurotrophic factor (GDNF, STEMCELL Technologies), and 100 nM retinoic acid (Tocris).

### Growth kinetics in human spinal cord organoids

3-DiSC hSCOs were inoculated with rEV-D68 at 14 days post-propagation in pools of 8–12. Viruses were diluted to 10^5^ PFU per pool in N2B27 medium. Pools were either infected with the indicated strain or a mock inoculum of PBS. Virus was adsorbed on hSCOs for 1 h at room temperature with rocking. After 1 h, the inoculum was aspirated, and the organoids were washed three times with PBS without calcium/magnesium (HyClone). Pools were transferred to new plastic to ensure there was no residual inoculum present, and fresh growth medium was added. hSCOs were incubated at 33°C, 5% CO_2_ for the duration of the experiment. Supernatants were taken at 0, 24, 48, 72, and 96 hpi and stored at −80°C. At 96 hpi, hSCO were fixed with 4% paraformaldehyde in PBS (ThermoFisher) for immunofluorescent staining as indicated.

### Viral titers

Infectious virus was quantified either by standard plaque assay or 50% tissue culture infectious dose assay (TCID_50_) on RD cells, as indicated. TCID_50_ titers were enumerated by the Spearman-Kärber method (49).

### Immunofluorescence staining

Immunofluorescence staining on fixed hSCOs was completed as previously described (50). Briefly, hSCOs were washed with 1% PBS-BSA and PBS-T at 4°C. The remaining washes and antibody dilutions were in organoid wash buffer (0.1% Triton X-100 [vol/vol], 0.2% BSA [mass/vol] in PBS). Primary antibodies VP1 (Genetex, GTX132313) and Tuj-1 (ThermoFisher, 480011) were used at a 1:250 dilution. Secondary antibodies Alexa Fluor 647 (Thermo Fisher, A-21245) and Alexa Fluor 488 (A-11001) were used at a 1:1,000 dilution. DAPI (4′,6-diamidino-2-phenylindole, dihydrochloride) (ThermoFisher, D1306) was used at a 1:300 dilution. hSCOs were cleared for 30 min with 60% glycerol in 2.5 M fructose before mounting.

### Confocal imaging

Microscope slides were imaged on a Leica Stellaris 5 confocal microscope equipped with a pulsed white light laser as an excitation source and an acousto-optical beam splitter and Leica Hybrid Detectors. Volumetric Z-stacks were imaged with a 20× objective. Sequential scanning was used with 2× line averaging by frame. The following parameters were used: dsDNA was measured at 403 nm, Tuj1 was measured at 488 nm, and VP1 was measured at 647 nm. Images were processed with FIJI 2 version 2.9.0.

### Statistical analysis

A Kruskal-Wallis test was performed in R (version 4.4.1) to select appropriate statistical tests for each data set, and either a one-way ANOVA or Mann-Whitney *U*-test was used, as indicated in each figure legend, to determine statistical significance. Linear regression was used to analyze the relationship between the ratio of T7 polymerase to cDNA infectious clone and viral titer and to evaluate differences in replication kinetics between viral strains.

## Data Availability

The following GenBank accession numbers were used to source VP1 sequences of each strain in this study: NC_038308 (USA/Fermon), MN240506 (USA/WI/2009-23230), KM851230 (USA/IL/2014-18952), MN245982 (USA/OH/2018-23088), MN246015 (USA/IL/2018-23252), and KX675261 (USA/FL/2016-19504). EV-D68 cDNA infectious clones were obtained through BEI Resources, National Institute of Allergy and Infectious Diseases (NIAID), National Institutes of Health (NIH). Requests for reagents or protocols should be directed to the corresponding author.
